# “Exercising hard or hardly exercising?” Objective and subjective measures of physical activity in patients with anorexia nervosa

**DOI:** 10.1186/2050-2974-3-S1-O55

**Published:** 2015-11-23

**Authors:** Sarah Young, Paul Rhodes, Stephen Touyz, Jon Arcelus, Caroline Meyer, Kathleen Pike, Sloane Madden, Ross Crosby, Evelyn Attia, Stacy Clemes, Phillipa Hay

**Affiliations:** 1 University of Sydney, Sydney, Australia; 2 Loughborough University, Loughborough, UK; 3 University of Warwick, Coventry, UK; 4 Columbia University, New York, NY, USA; 5 Children's Hospital at Westmead, Sydney, Australia; 6 Neuropsychiatric Research Institute, Fargo, ND, USA; 7 Western Sydney University, Sydney, Australia

## 

Compulsive exercise in anorexia nervosa (AN) has been associated with adverse effects in treatment, and is a major risk factor for relapse. However, exercise is often surreptitious and measurement problematic. For example, females in community samples have been found to over-report physical activity when compared to objective assessment. The current study aimed to explore the relationships between objectively recorded and subjectively reported physical activity (PA) in AN outpatients. Participants were 34 females with AN enrolled in a randomised-controlled trial of outpatient treatment of anorexia nervosa (Hay et al., in progress). They completed the Eating Disorder Examination (EDE) interview; self-report questionnaires assessing eating disorder and exercise cognitions and behaviours; and wore an accelerometer for 4 days as an objective PA measure. Participants also self-reported their average daily PA. Results demonstrated significant under-reporting (objective PA greater than self-report PA) on light and total PA, but not vigorous or moderate PA. Discrepancy scores between moderate PA (accelerometer – self-report) were also negatively correlated with motivation to change in AN (p<0.05). Clinically, it is important that professionals are aware of such discrepancies between methods, as well as the limitations of accelerometer devices and self-report measures.

